# Expression of ErbB3-Binding Protein-1 (EBP1) during Primordial Follicle Formation: Role of Estradiol-17ß

**DOI:** 10.1371/journal.pone.0067068

**Published:** 2013-06-20

**Authors:** Anindit Mukherjee, Shyamal K. Roy

**Affiliations:** 1 Departments of Cellular and Integrative Physiology, University of Nebraska Medical Center, Omaha, Nebraska, United States of America; 2 Department of Obstetrics and Gynecology, and Olson Center for Women’s Health, University of Nebraska Medical Center, Omaha, Nebraska, United States of America; Baylor College of Medicine, United States of America

## Abstract

The formation of primordial follicles involves the interaction between the oocytes and surrounding somatic cells, which differentiate into granulosa cells. Estradiol-17ß (E) promotes primordial follicle formation *in vivo* and *in vitro*; however, the underlying mechanisms are poorly understood. The expression of an ERBB3-binding protein 1 (EBP1) is downregulated in 8-day old hamster ovaries concurrent with the increase in serum estradiol levels and the formation of primordial follicles. The objectives of the present study were to determine the spatio-temporal expression and putative E regulation of EBP1 in ovarian cells during perinatal development with respect to primordial follicle formation. Hamster EBP1 nucleic acid and amino acid sequences were more than 93% and 98% similar, respectively, to those of mouse and human, and contained nucleolar localization signal, RNA-binding domain and several phosphorylation sites. EBP1 protein was present in somatic cells and oocytes from E15, and declined in oocytes by P1 and in somatic cells by P5. Thereafter, EBP1 expression increased through P7 with a transient decline on P8 primarily in interstitial cells. EBP1 mRNA levels mirrored protein expression pattern. E treatment on P1 and P4 upregulated EBP1 expression by P8 whereas E treatment on P4 downregulated it by 72 h suggesting a compensatory upregulation due to E pretreatment. Treatment with an FSH-antiserum, which suppressed primordial follicle formation, prevented the decline in EBP1 levels, and the effect was reversed by E treatment. Therefore, the results provide the first evidence that EBP1 may play an important role in mediating the effect of E in the differentiation of somatic cells into granulosa cells during primordial follicle formation.

## Introduction

Undifferentiated somatic cells surrounding the egg nests differentiate into pregranulosa cells, and invade the egg nest to encircle individual oocytes forming primordial follicles. Pregranulosa cells become granulosa cells once primordial follicles are formed, and this represents the first critical morphogenetic process in folliculogenesis. Defect in primordial follicle formation may lead to premature ovarian failure and infertility [1]–[3]. The regulation of primordial follicle morphogenesis is not well understood. Deletion of GDF9 [4], [5] or BMP15 [6], [7], or inactivation of endogenous FSH [8], [9] adversely affects primordial follicle formation. Estradiol-17ß (E), at physiological dose level, supports follicle formation [10]–[14]. E as well as ESR1 and ESR2 levels increase during primordial follicle formation *in vivo* [13]–[15]. E promotes primordial follicle formation *in vivo* and *in vitro.* However the mediators of E action are yet to be identified. Proteomic analysis of developing hamster ovaries has identified ERBB3 binding protein 1 (EBP1) to be significantly downregulated in hamster ovaries on postnatal day 8 (P8) when morphologically distinct primordial follicles can be first identified [16].

EBP1, the human homolog of PA2G4 family of proteins, is transcribed from the gene PA2G4 [17]. EBP1 has a 48 kDa and a 42 kDa isoforms; however, the 48 kDa isoform predominates in mammals, including the hamster [16]. EBP1 is highly conserved in eukaryotes, ubiquitously expressed [18] in tissues and structurally similar to methionine aminopeptidase [19], [20]. It plays an important role in cell proliferation and differentiation [20]–[22]. Binding of EBP1 to the 15 amino acid juxtamembrane domain of ERBB3 [23] via its 1–105 amino acid region at the N-terminal end [24] is regulated by PKC. Heregulin phosphorylates EBP1 at ser-360 causing it to dissociate from ERBB3 [23]. Phosphorylated EBP1 associates with phosphorylated AKT [25] as well as migrates to the nucleus [23]. Overexpression of EBP1 leads to cell cycle arrest [26] in breast cancer cells and fibroblasts [20]. The tumor suppressor activity of EBP1 has been ascribed to its ability to translocate to the nucleolus [27]. Deletion of EBP1 in mice results in subfertility with more than 50% decrease in the litter size than their wild type or heterozygous counterpart [28] suggesting that EBP1 may potentially play an important role in ovarian function. However, virtually nothing is known about the role of EBP1 in ovarian follicular development, including primordial follicle formation or its regulation by E during primordial follicle formation in the hamster. Therefore, as a first step towards understanding the role of EBP1 in primordial folliculogenesis, the spatio-temporal expression of EBP1 transcript and protein in ovarian cells with respect to primordial follicle formation and its regulation by E were evaluated in the present study.

## Materials and Methods

Animal experimentation was done according to the United States Department of Agriculture (USDA) and University of Nebraska Medical Center Animal Care and Use Committee (Institutional Animal Care and Use Committee, IACUC) guidelines. Adult golden male and female hamsters were purchased from Harlan Laboratories (Madison, WI) and maintained in a climate-controlled room with 14 h light and 10 h dark with free access to food and water according to the IACUC and the USDA guidelines. University of Nebraska Medical Center Animal Care and Use Committee approved the use of hamsters for this study (Permit number: 93-080-04). Females with at least three consecutive estrous cycles were mated with males on the evening of proestrus, and the presence of sperm in the vagina next morning was considered Day 1 of pregnancy. Hamster gestation lasts for 16 days, and pups are born on 16^th^ day of gestation, which was considered as the 1^st^ day of postnatal life.

Estradiol-17ß cypionate (E) was purchased from Pfizer company (Kalamazoo, MI), phenol red-free Dulbecco’s modified Eagle medium (DMEM) was purchased from Life Tehnologies, Inc. (Carlsbad, CA), linolenic acid, bovine serum albumin for tissue culture and monoclonal ß-tubulin antibody were purchased from Sigma Chemical Company (St. Louis, MO), human holo-transferrin, selenium and bovine insulin were purchased from Collaborative research (Bedford, MA), Falcon non-tissue culture inserts and plates, solvents for histology, Western blotting supplies and other fine chemicals were purchased from Thermo-Fisher and GE Biosciences (Pittsburgh, PA) and, plastic embedding medium was from Electron Microscopy Sciences (Hatfield, PA), RNA extraction and quantitative RT-PCR (qPCR) chemicals were from Qiagen (Valencia, CA), primers and fluorescence-labeled probes were from Eurofins (Huntsville, AL), the polyclonal EBP1 antibody was purchased from Millipore Corporation (Billerica, MA), HRP-conjugated and DyLight fluorescence-tagged second antibodies were from Jackson Immunoresearch (West Grove, PA).

### Partial Cloning of Hamster EBP1 cDNA

Because nucleic acid sequence information for hamster EBP1 was unavailable, we wanted to partially clone the hamster EBP1 cDNA from ovarian RNA using reverse transcription and PCR. Total ovarian RNA was isolated from proestrus hamsters using RNeasy mini kit as per manufacturer’s instruction. The sequences of forward and reverse primer sequences were: 5′-GAGCAGCAGGAGCAAACTATC-3′ and 5′-CCCAGCTCCATTCTCTTCTAAG-3′, respectively, corresponding to 1164 bp mouse pa2g4 gene (NM_011119). The primers were designed using vector NTI (Life Technologies, Inc.) primer design software version 11. The conditions for PCR was similar to that described previously for ESR1 [29] with an annealing temperature of 55°C. After verifying the predicted size of the cDNA by gel electrophoresis, EBP1 cDNA was cloned into PCR4TOPO vector (Invitrogen), transformed into Top10 cells, and sequenced (University of Nebraska at Lincoln DNA Core facility). The similarity of hamster EBP1 cDNA sequence was compared with available sequences in the genebank (National Institute of Health, Bethesda, MD) and partial amino acid sequence was deduced. The sequence was deposited at the National Center for Biotechnology Information (NCBI) genebank database (Bethesda, MD).

### Quantitation of EBP1 mRNA Levels in Hamster Ovaries during Perinatal Development

Total RNA was isolated from embryonic day 15 (E15) through postnatal day 8 (P8) hamster ovaries using Picopure kit (company, location) as per manufacturer’s instruction. The levels of EBP1 mRNA in 1 µg ovarian RNA was quantified by qPCR using a standard curve made with increasing amount of EBP1 cDNA, qPCR primers and 6-FAM labeled Taqman probe in Opticon 2 thermocycler (Bio-Rad, CA). The sequences of forward and reverse primers, and probe were:

5′-TCTTTGTGCTGAAGCTGCCTTA-3′, 5′-TGTTCCAGGCTTCTGTCACTTG-3′, and 5′-ACCGGTCAAACCCGGAAACCAGAACAC-3′, respectively. The annealing temperature for qPCR was 55°C.

### Western Blot Analysis of EBP1 Levels in Hamster Ovaries during Perinatal Development

Ovaries from E15 through P10 hamsters were sonicated in lysis buffer (10 mM Tris-HCl, pH 7.4; 100 mM NaCl; 1 mM EDTA; 1 mM EGTA; 1 mM NaF; 20 mM Na_4_P_2_O_7_; 1% Triton X-100; 10% glycerol; 0.1% sodium dodecyl sulfate; and 0.5% deoxycholate) containing a protease inhibitor cocktail and phenylmethylsulfonylfluoride (PMSF) at 4°C. Forty-µg protein was fractionated in denaturing reducing polyacrylamide gels, electrotransferred to Optitran nitrocellulose membrane, and the membrane was treated with an enhancing solution (Pierce). After blocking, the membrane was probed with 1∶2000 dilution of the anti-EBP1 or anti-ß-tubulin (TUBB) antibody and an appropriate second antibody. The chemiluminescence signal was developed using the ECL Advance (GE Healthcare), captured by a UVP gel documentation system (UVP, Upland, CA) and the pixel density of the band was digitized. Digitized values for EBP1 were normalized against tubulin and presented as a ratio. Each group had at least three or more replicates of samples collected from three different animals.

### Immunofluorescence Localization of EBP1 and MVH

Ovaries were collected from E15 through P8 hamsters and frozen in OCT (Optimum Cutting Temperature) compound on LN2 cooled methyl-butane as previously described [16]. Frozen sections at 6 µm were fixed in freshly prepared ice-cold 4% paraformaldehyde in PBS (pH 7.4) for 10 min and stained for EBP1 protein as described previously [16]. The images were captured by the Openlab (Perkin-Elmer, Waltham, MA) image analysis software. The exposure time of the camera was set for subtracting background fluorescence that was present in sections incubated with the non-immune IgG of the host species. EBP1 specific fluorescence immunosignal was merged with the nuclear signal DAPI (4′, 6-diamidino-2-phenylindole) to determine the cellular site of protein expression. The nuclear signal was blue, whereas the EBP1 signal was green. The expression of the mouse vasa homolog (MVH; red fluorescence) in the oocytes of P4 and P8 ovaries was also detected to distinguish them from undifferentiated somatic cells (before P8) and newly formed granulosa cells (after P8).

### Effect of Estradiol-17ß (E) on Ovarian EBP1 mRNA and Protein Expression during Primordial Follicle Formation

Hamster pups were injected sc with 1 µg estradiol cipionate (E) in 20 µl sesame oil on P1 and P4. Ovaries were collected at different time following E injections and processed for EBP1 mRNA and protein analysis. In a separate experiment, the endogenous FSH was neutralized by injecting pregnant hamsters sc, with 200 µl of an anti-FSH serum on 12^th^ day of gestation as described previously [8]. After birth, pups received sc injection of 1 µg E in 20 µl sesame oil on P1, or on P1 and P4. Ovaries were collected on P8 in ice cold PBS for immunoblot analysis of EBP1 or tubulin, or frozen in OCT for immunofluorescence localization of ESR1. Sera were saved for E RIA as described earlier [30]. All samples were assayed simultaneously to avoid inter-assay variation. The intra-assay variation was 5%.

### Statistical Analysis of Data

All experiments were repeated at least three times using ovaries obtained at different times. Quantitative data were analyzed by one-way ANOVA coupled with the Newman-Keuls *post hoc* test using GraphPad Prism 5 software (Graph Pad software Inc., La Jolla, CA). The level of significance was 5%.

## Results

### Cloning of Hamster EBP1

**Figure 1:**
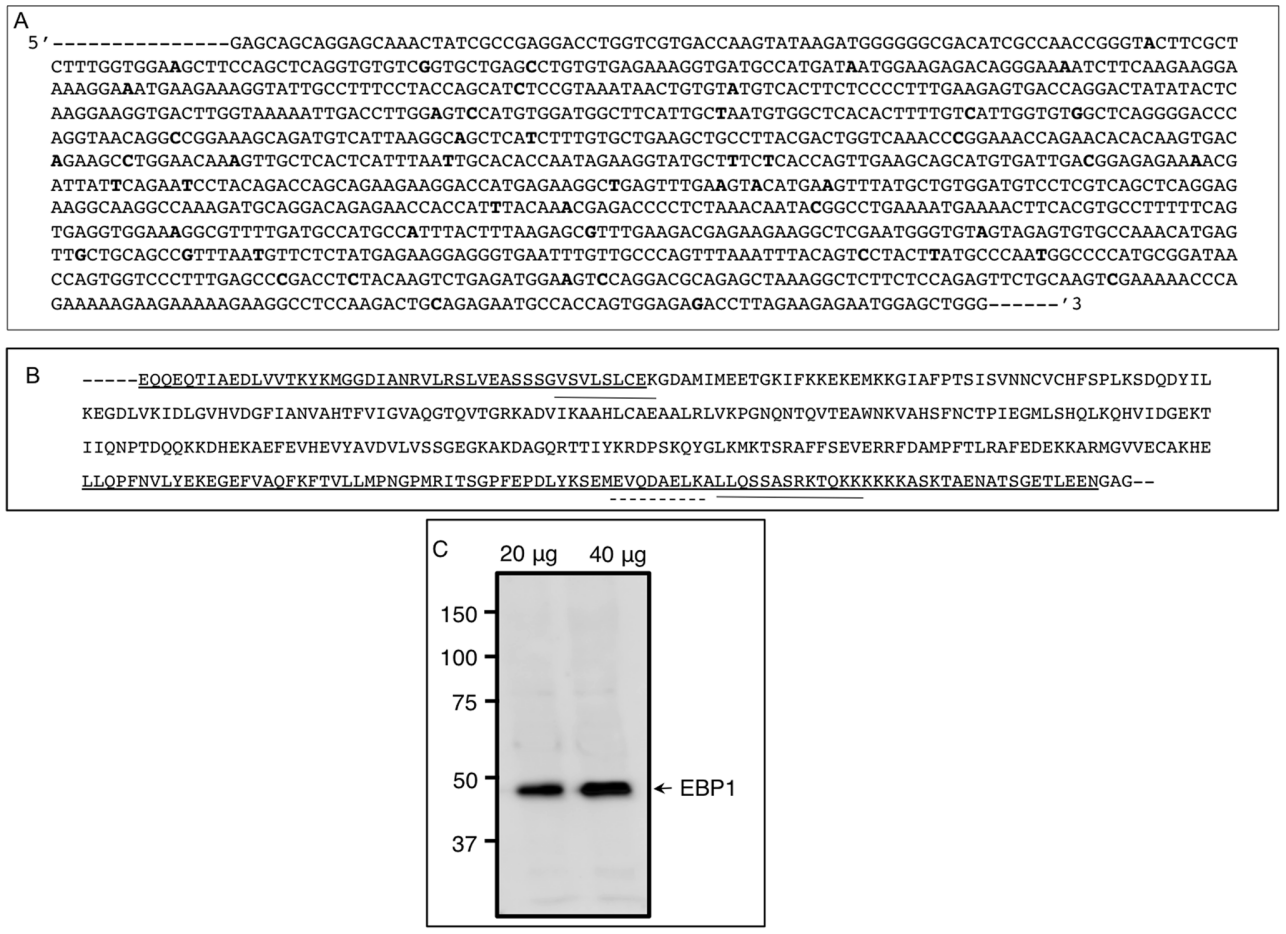
Nucleic acid and amino acid sequence of hamster EBP1. (A) Partial nucleic acid sequence of the hamster EBP1 cDNA. Hamster EBP1 was around 95% similar to that of the other species. Bold face letters indicates nucleotide differences with that of the mouse EBP1. (B) Deduced amino acid sequence of the hamster EBP1 highlighting nucleolar localization signal (underline), RNA binding motifs (overline) and a transcriptional co-activator/repressor binding site (dashed overline). (C) A Western blot validating the specificity of the polyclonal EBP1 antibody. The antibody detected one approximately 48 kDa protein band in two different amounts of hamster ovarian total protein. The increase in chemiluminescence signal with higher amounts of ovarian protein indicated non-saturation of the antibody used in the analysis.

The rationale for cloning the hamster EBP1 cDNA was to determine the similarities and differences between model rodent species and human, to generate hamster-specific qPCR primers and probe, and nucleic acid reagents for further study. PCR generated a predicted 1164 bp EBP1 cDNA (Fig. 1A), which was 95%, 94%, and 93% similar to that of the mouse (NM_011119.3), rat (NM_001004206.1), and human (NM_006191.2) EBP1 cDNAs, respectively. The deduced amino acid sequence (Fig. 1B) was 99% similar to the corresponding region of the mouse, rat and human EBP1, and contained a nucleolar localization signal, an RNA binding domain (UNIPROT ID_ Q9UQ80) as well as the predicted α10 helix domain, which allows the protein to bind to transcriptional repressors/coactivators. Hamster EBP1 cDNA sequence was submitted to Genebank (accession number HM851533). NetPhos analysis revealed eight potential threonine, twelve serine, and four tyrosine phosphorylation sites throughout the amino acid sequence. Protein kinase C, casein kinase II and protein kinase A appeared to be the preferred kinases for EBP1 phosphorylation (data not shown). As predicted by the manufacturer, the polyclonal EBP1 antibody detected one major band approximately at 48 kDa (Fig. 1C). Further, the antibody could detect EBP1 protein in as low as 20 µg ovarian total protein; however, for reproducibility, most of the analyses were done using at least 40 µg ovarian protein. These results not only validated the antibody for Western immunoblotting, but the presence of only one band also eliminated any possible non-specific immunosignal in immunofluorescence localization.

### Perinatal Expression of EBP1 mRNA and Protein

**Figure 2:**
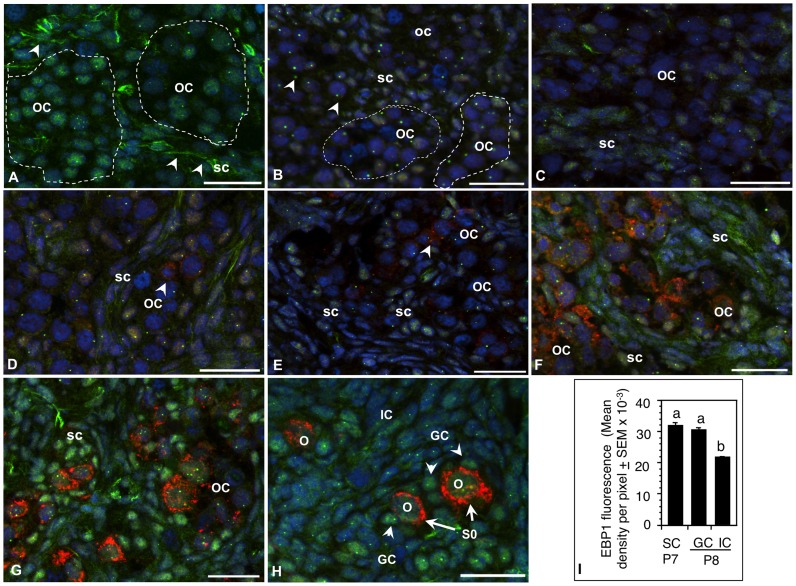
Spatiotemporal expression of EBP1 protein during perinatal ovary development in the hamster. EBP1 (green) was localized in germ cells as well as undifferentiated somatic cells in the fetal ovary; however, the cell-type specific location and the degree of expression shifted to somatic cells as the ovary developed. Sections of (A) an E15, (B) a P1, (C) P3, (D) P4, (E) P5, (F) P6, (G) P7, and (H) P8 ovaries. MVH expression (red) was detectable in the oocytes in P4 ovaries (D, arrowhead) and increased appreciably with the growth of the oocytes from P6 (F) through P8 (H). Nuclei were stained with DAPI. Germ cell nests or oocyte clusters (OC) were demarcated by broken line. SC, somatic cells, O, oocytes, S0, primordial follicles, scale bar = 10 µm. (I) Quantitative representation of EBP1 immunosignal in undifferentiated somatic cells of P7 ovaries and in the granulosa cells (GC) of primordial follicles and interstitial cells (IC) of P8 ovaries. Bars with different letter = P<0.05.

The rationale was to delineate whether EBP1 mRNA and protein expression in the ovary altered during postnatal development with respect to primordial follicle formation. EBP1 was localized in ovarian cells as early as E15, but spatial localization changed with development (Fig. 2). EBP1 was present in the nucleus of primordial oocytes in the egg-nests as well as in the somatic cells of E15 ovaries; however, distinct cytoplasmic/membrane-associated localization was evident in somatic cells surrounding the egg nests (Fig. 2A, arrowheads). Further, nucleolar staining was localized only in the oocytes (Fig. 2A). EBP1 immunosignal declined both in the oocytes and somatic cells on P1, but the decrease in the oocytes was more prominent (Fig. 2B). Cytoplasmic localization was difficult to ascertain because of the compact nature of somatic cells. Nevertheless, distinct nucleolar localization was evident in the oocytes as well as in undifferentiated somatic cells (Fig. 2B). EBP1 expression remained low through P5 (Figs. 2C–E). MVH expression in the oocytes was visible by P4 (Fig. 2D, arrowhead) and increased thereafter (Fig. 2H). By P6, the intensity of EBP1 immunosignal increased noticeably only in somatic cells surrounding the egg nests (Fig. 2F), but cells elsewhere in the ovary did not show appreciable change (data not shown). Further, diffused cytoplasmic immunosignal was visible (Fig. 2F). EBP1 expression increased further on P7 with staining localized in the nucleus of somatic cells adjacent to egg nests (Fig. 2G). Cells elsewhere in the ovary showed modest change. Oocytes had modest immunosignal with visible nucleolar localization (Fig. 2G). Whereas newly formed granulosa cells (GC) of primordial follicles (S0) retained strong EBP1 staining (Figs. 2H, arrowheads and 2I) similar to that observed for the somatic cells adjacent to the oocytes on P7, the intensity of EBP1 in interstitial cells decreased further by P8 (Figs. 2G and 2I). Punctate nucleolar staining was evident in all cell types (Fig. 2H). The first cohort of primordial follicles appeared on P8 [8].

**Figure 3:**
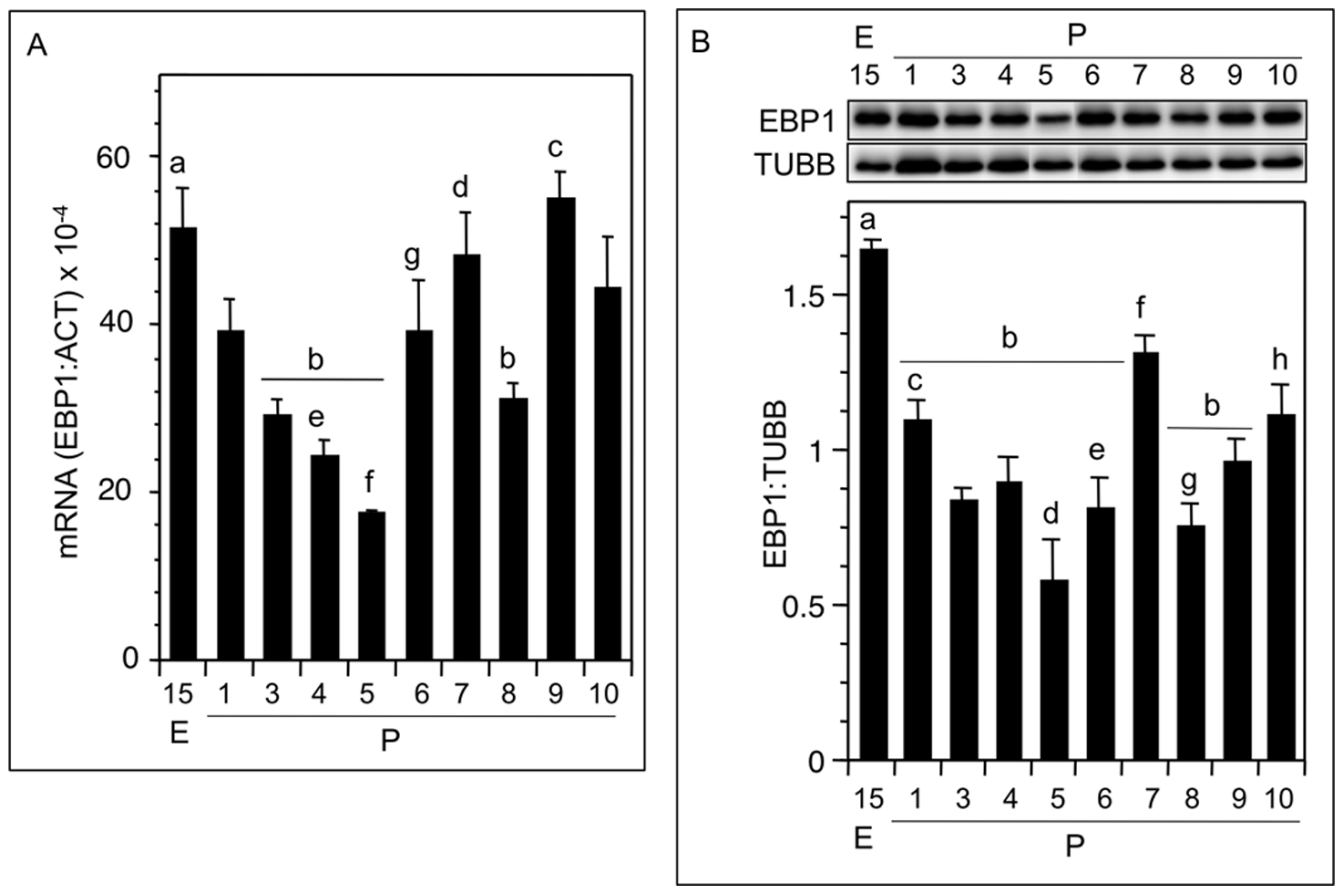
EBP1 expression pattern during perinatal ovary development in the hamster. (A) EBP1 mRNA expression during perinatal ovary development. Each bar represented a ratio of EBP1: ACT mRNA. Note a marked decline in EBP1 mRNA by P5. (B) Representative Western blot of EBP1 protein expression during perinatal hamster ovary development (top), and ratio of densitometric quantification of EBP1 and TUBB bands in the Western blots (bottom). Each bar in A or B reflected a mean±SEM of at least 3 or 5, respectively, ovary samples from different animals. A: b vs a, c, P<0.05; d vs e, f, P<0.05; g vs f, P<0.05; B: a vs b, h, P<0.05; d vs c, f, h, P<0.05; f vs e, g, P<0.05. E, embryonic age, P, postnatal age.

Consistent with the immunofluorescence findings, EBP1 mRNA levels decreased significantly from E15 through P5, and then reversed by P6; however, the values did not exceed those observed for P1 (Fig. 3A). EBP1 transcript levels dropped significantly by P8 compared to those of the P7 before increasing again on P9 (Fig. 3A). The pattern of EBP1 mRNA expression was generally reflected in the EBP1 protein levels (Fig. 3B). The increase in EBP1 mRNA correlated with slight but significant rise in EBP1 protein on P6, but the total EBP1 protein levels were not different from those observed on P1 (Fig. 3B).

### Effect of Estradiol-17ß (E) on EBP1 mRNA and Protein Expression in Postnatal Hamster Ovaries

**Figure 4:**
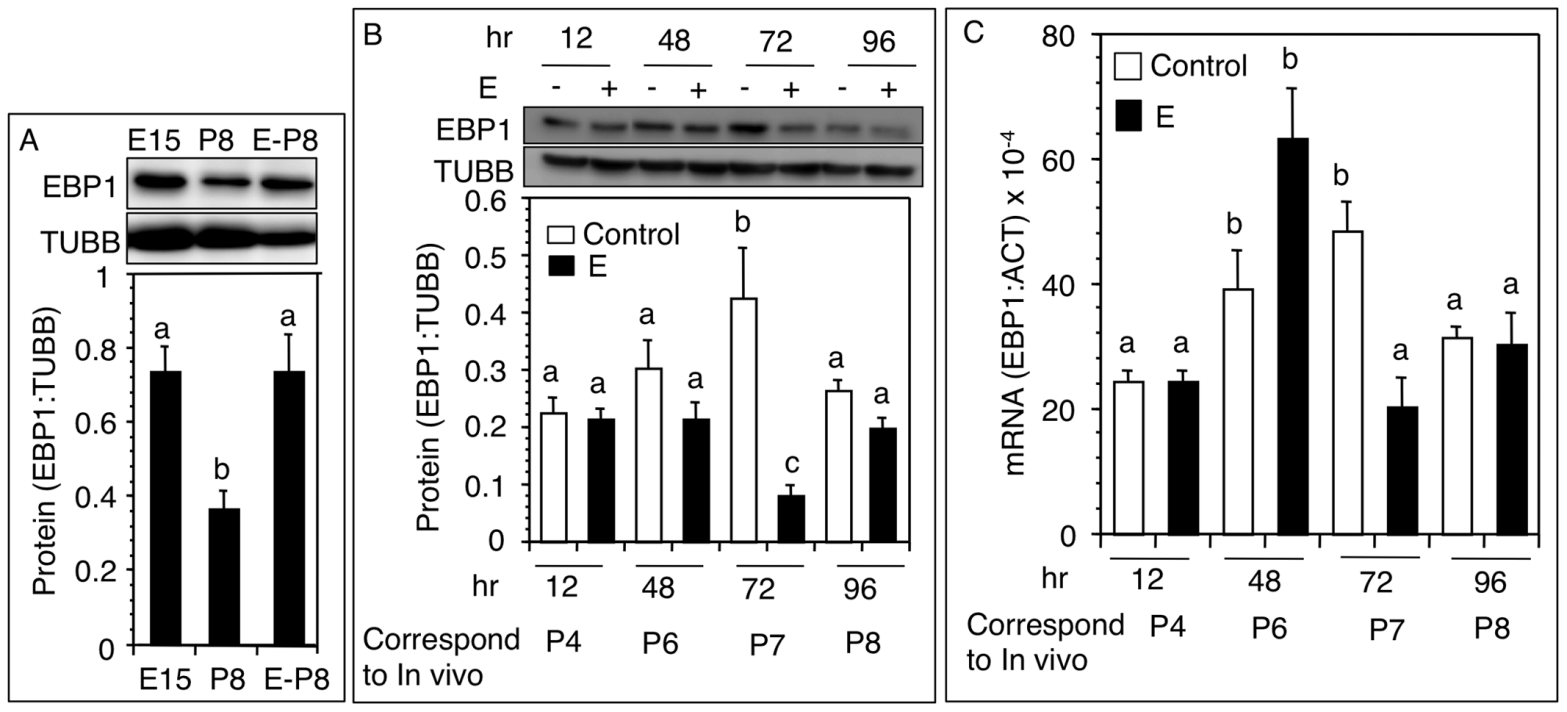
Changes in ovarian EBP1 protein and mRNA levels following estradiol (E) treatment in vivo. (A) Western blot analysis (top) and ratio of EBP1: TUBB (bottom) levels in E15 ovaries, and P8 ovaries from hamsters injected with vehicle or 1 µg E on P1 and P4. (B) Time course effect of E on ovarian EBP1 protein expression. P4 hamsters were injected with a single 1 µg dose of E, and ovaries retrieved at specified time after the injection. Control ovaries were collected in parallel from vehicle treated hamsters corresponding to days of development *in vivo*. (C) Time course effect of E on ovarian EBP1 mRNA expression (EBP1: ACT) in P4 hamsters treated in vivo with a single 1 µg dose of E. Control ovaries were collected in parallel from vehicle treated hamsters corresponding to days of development in vivo. Each bar represents a mean ± SEM of at least 3 different animals. Bars with different letter = P<0.05. E, embryonic age, P, postnatal age.

The rationale was to investigate whether the decrease in EBP1 levels on P8 was caused by E. Serum E increased corresponding to the formation of primordial follicles [13]. EBP1 levels in untreated hamsters declined significantly on P8 compared to E15; however, E2 treatment on P1 and P4 upregulated EBP1 expression in P8 in hamsters (Fig. 4A). To determine if the suppressive effect of E was transient resulting in compensatory increases in EBP1 levels we examined the temporal effect of E on EBP1 expression after a single dose of E on P4. As observed in developing hamsters (Fig. 3A), EBP1 levels increased from P4 through P7 before declining by P8 (Fig. 4B). In E-treated ovaries, EBP1 protein levels remained unchanged up to 48h followed by a significant decrease by 72 h compared to time-matched untreated ovaries (Fig. 4B). EBP1 levels in E-treated ovaries rebound to the levels of normal P8 by 96 h (Fig. 4B). EBP1 mRNA levels increased markedly by 48 h after E treatment, and then declined sharply by 72 h (Fig. 4C).

### Effect of Estradiol-17ß (E) Replacement in FSH Antiserum–treated Hamsters on Ovarian EBP1 Expression

**Figure 5:**
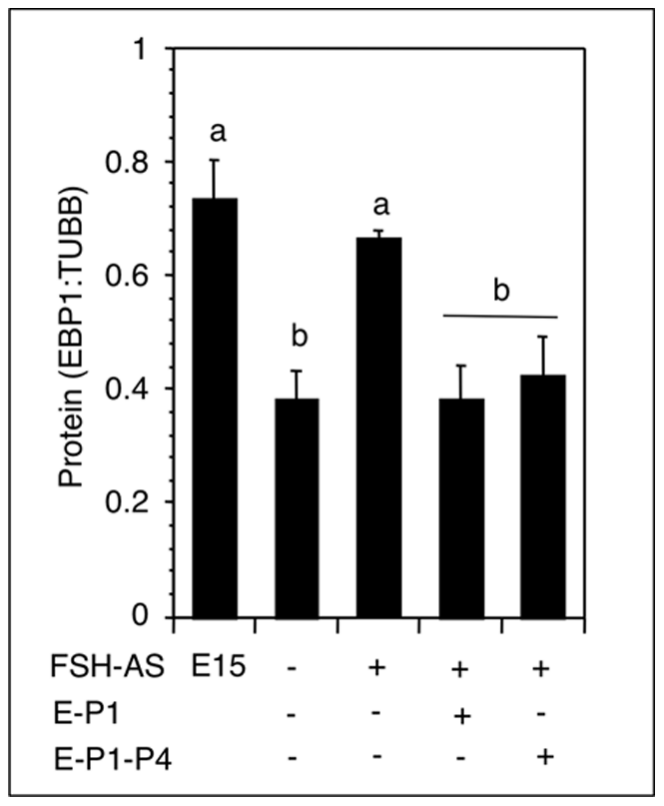
EBP1 protein expression in hamsters exposed in utero to the FSH-antiserum on E12. Pups were injected once/day with a sc dose of 1 µg E or 20 µl sesame oil (vehicle) either on P1 or on P1 and P4. Ovaries were retrieved on P8, and ovarian EBP1 and TUBB protein levels were examined by Western blotting. Each bar represented a mean ± SEM of at least 4 hamsters. Bars with different letters = P<0.05. E, embryonic age, P, postnatal age.

In utero treatment with an FSH-antiserum causes serum FSH levels to be undetectable [8]. Consistent with FSH unavailability serum E was undetectable (data not shown). FSH stimulates E production by prenatal hamster ovaries in vitro [13]. Therefore, we hypothesized that FSH-antiserum treatment should prevent ovarian EBP1 decline on P8, but E would be able to reinstate the decline. Whereas ovarian EBP1 levels in untreated P8 hamsters declined significantly concurrent with increased serum E levels [13], EBP1 levels remained high in hamsters treated in utero with an FSH-antiserum (Fig. 5). However, administration of E either on P1 or both on P1 and P4 reversed the antiserum effect and significant downregulation of EBP1 levels was evident (Fig. 5).

## Discussion

The results of this study provide the first evidence that EBP1, an ERBB3 binding protein, is expressed in perinatal ovary cells and the expression is downregulated in ovarian somatic cells with their differentiation into early granulosa cells of primordial follicles. The presence of only one 48 kDa band in ovarian homogenate corroborates with the results furnished by the manufacturer and validates the specificity of the antibody in detecting EBP1 in the immunoblots. Therefore, it stands to reason that fluorescence generated by the EBP1 antibody in immunolocalization reflects EBP1 antigen in ovarian cells. Because purified EBP1 protein is unavailable, additional validation of the antibody cannot be done. E regulates EBP1 turnover in developing ovary cells. The high degree of sequence similarity of hamster EBP1 cDNA and amino acid sequences with those of other mammals including the human suggests that this protein is highly conserved across species and also indicates its evolutionary functional importance. EBP1 amino acid sequence contains nucleolar localization sequence, which correlates with punctate EBP1 localization in the nucleolus of ovarian cells. EBP1 also contains a RNA binding site as well as the alpha10 helix sequence, which bind to the transcriptional co-activators or repressors [31], [32]. Potential serine, tyrosine and threonine phosphorylation sites suggest that specific phosphorylation of EBP1 may affect its function. It has been shown that phosphorylation of EBP1 at serine 363 results in its exclusive nuclear localization, and mutation of serine 363 to alanine significantly decreases the ability of EBP1 to repress transcription and suppress cell proliferation [33]. Similarly, phosphorylation of EBP1 at threonine 261 by p21-regulated serine/threonine kinase, PAK1, in MCF-7 breast cancer cell line results in suppression of EBP1 transcriptional activity, reduction in ErbB2 protein levels and induction of tamoxifen resistance, but a threonine to alanine mutation reverses the effect [34].

The absence of an increase in total ovarian EBP1 protein levels despite increases in EBP1 immunostaining on P6 reflects a localized rather than a global increase in EBP1 expression corresponding to primordial follicle formation. The temporal changes in EBP1 expression and shift in intracellular localization during perinatal ovary development suggest a potentially important role of EBP1 in primordial follicle formation. Evidence indicates that shift in intracellular localization of EBP1 is linked to mitogenic and migratory activities of cells [33]. Further, heregulin binding to ErbB3 results in the dissociation of EBP1, which binds to nuclear AKT [23] and suppresses caspase-activated DNase to prevent DNA fragmentation. Our preliminary data suggest EBP1 and ErbB3 colocalize in the somatic cells of perinatal hamster ovaries and heregulin exposure of P6 ovarian cells in culture results in ERK1 phosphorylation (data not shown). Whether activation of ErbB3 by heregulin results in EBP1 dissociation or ERK1 activation leads to EBP1 phosphorylation in ovarian somatic cells is under study. The downregulation of ovarian EBP1 expression by P8 following the endogenous rise in E by P6 [13] or by 72 h after exogenously administered E on P4 may be ascribed to the availability of the estrogen receptor-alpha (ESR1). ESR1 expression increases gradually during this period of neonatal development [15] under gradual increase in serum E levels [13]. The increase in EBP1 mRNA levels without a concurrent rise in EBP1 protein levels at 48 h after E treatment suggests a potential block in translation, which may also be responsible for reduced levels of ovarian EBP1 by 72 h. Nevertheless, the results prove that E downregulates ovarian EBP1 expression. We have shown that a single injection of 1 µg E on P1 raises serum E level to 200 pg/ml [13] on P8. We speculate that ESR1 may become refractory due to chronic high levels of E beyond 72 h, resulting in a rebound in EBP1 expression as evident in Fig. 4A. This conjecture is supported by the finding that reduced expression of ESR1 in P8 hamster ovaries exposed *in utero* to the FSH antiserum [15] corresponds to higher expression of EBP1 on P8. In prostate cancer cells, EBP1 has been shown to suppress translation of androgen receptor mRNA [35]. The increase in EBP1 levels with corresponding decrease in ESR1 in ovarian cells deprived of FSH in vivo leads us to speculate that EBP1 may use a similar mechanism in developing ovarian cells to regulate ESR1 expression. E, by transiently downregulating EBP1, promotes ESR1-mediated biological effects. The sustained low levels of ovarian EBP1 in P8 hamsters exposed *in utero* to FSH-antiserum reflects altered ovarian environment in FSH-antiserum-treated hamsters. Although the results of the present study suggest that EBP1 may function as a potential mediator of E effect on early follicular development, the exact role of EBP1 in ovarian follicular development, especially during PF formation is not yet known. EBP1 deletion in mice results in more than 50% decrease in the litter size [28], thus indicating that EBP1 may possibly play a role in ovarian follicular development; however, the information about ovarian morphology of EBP1 null mice is not available. In a preliminary study, we have observed that knockdown of EBP1 in postnatal hamsters results in a block in the breakdown of egg nests and almost complete block in primordial follicle formation (data not shown). Therefore, it can be speculated that while transient downregulation of EBP1 may allow ErbB3 action, EBP1 prevents ErbB3 over action for orderly differentiation of ovarian somatic cells.

In summary, the results suggest that EBP1 may play an important role in the differentiation of ovarian somatic cells into granulosa cells by temporally regulating the effect of E. Therefore, it stands to reason that E may use this mechanism to regulate ErbB3 action in perinatal ovary cells during primordial folliculogenesis. The differential expression as well as spatial localization of EBP1 in ovary cells during primordial follicle formation suggests that EBP1 as a downstream regulatory protein may modulate the effect of proteins that play critical role in the differentiation of ovarian somatic cells. Further, E regulation of EBP1 expression depends on the availability of ESR1 and hormonal priming of ovarian cells. We speculate that EBP1 may also regulate ESR1 levels in differentiating ovarian somatic cells.
